# No interface energy barrier and increased surface pinning in low temperature baked niobium

**DOI:** 10.1038/s41598-022-09023-0

**Published:** 2022-04-01

**Authors:** Daniel Andrew Turner, Graeme Burt, Tobias Junginger

**Affiliations:** 1 Cockcroft institute, STFC Daresbury Laboratory, Warrington, WA4 4AD UK; 2 Lancaster University, Engineering, Lancaster, LA1 4YW UK; 3 Department of Physics, University of Victoria, Victoria, BC V8P 5C2 Canada; 4 TRIUMF, Accelerator division, Vancouver, BC V6T 2A3 Canada

**Keywords:** Materials science, Physics

## Abstract

Superconducting Radio-Frequency cavities are currently made out of niobium. Niobium cavities are limited by the magnetic field on the cavity walls due to the entry of vortices at the field of first vortex penetration, H$_{vp}$. Low temperature baking in vacuum or low pressure gas atmosphere removes the strong decrease of the quality factor with accelerating gradient (high field Q-slope). Some cavities reach surface magnetic field above the lower critical field H$_{c1}$. One hypothesis for this performance increase is that the outer layer affected by the treatments acts as a barrier for vortex penetration (effective bilayer). Using a vibrating sample magnetometer the field of first flux penetration (H$_{vp}$) was measured for Nb ellipsoids with various low temperature treatments. All H$_{vp}$ values were found to be consistent with the lower critical field, H$_{c1}$, as predicted for clean niobium. This led to the conclusion that a metastable flux free state above H$_{c1}$ cannot be observed in DC magnetometry for low temperature baked niobium unlike for bilayers consisting of two superconductors as previously published. The effect of flux pinning differed significantly between treatments, suggesting that the high field Q-slope mitigation might be related to vortex pinning in the surface of the cavities.

## Introduction

Particle accelerators often use superconducting radio frequency (SRF) cavities to accelerate the particle beam. Large electromagnetic fields are produced when the RF power is applied close to the resonant frequency of the cavity. The electric field generated accelerates the particle bunches as they pass through the cavity. The operating frequencies for SRF cavities typically range between 350 and 3900 MHz^1^ for elliptical multi-cell structures, with the optimum frequency being dependant on the application and the structure of the cavity. As a consequence of Maxwell’s equations a magnetic field is generated, which is proportional to the applied electric field. The magnetic field interacts with the cavity surface and is cancelled inside the superconductor by screening currents formed in the surface layer of the superconductor. The maximum accelerating gradient (E$_{acc}$) is limited by the magnetic field on the cavity walls. Superconductors experience field transitions at critical magnetic fields, which is a response to an externally applied field (H$_{ext}$). Below the lower critical field (H$_{c1}$) a type II superconductor will be in the Meissner state and behave as a perfect diamagnet. The field will be expelled from the superconductor due to screening currents which oppose the H$_{ext}$ such that H$_{ext}$ decays by 1/*e* over the London penetration depth ($\lambda _{L}$). Above H$_{c1}$ it becomes energetically favourable for vortices to be present within the superconductor. It is possible for a superconductor to remain in the Meissner state above H$_{c1}$ up to a superheating field (H$_{sh}$) due to the the Bean–Livingston surface barrier^2^. The Bean–Livingston barrier can be understood by considering a single vortex line at the surface of the superconductor. To fulfil the boundary condition at the surface an image vortex is introduced. This causes the vortex energy to depend on depth with an energy barrier for flux penetration present between H$_{c1}$ and H$_{sh}$. The superheating field (H$_{sh}$) is the field at which the energy barrier vanishes. Defects in the superconductor can act as nucleation sites. Therefore, it is assumed that only defect free superconductors can reach a metastable superheated state above H$_{c1}$. It has been argued that RF cavities can potentially remain in a metastable state above H$_{c1}$ if the time required to nucleate fluxoids is long compared to the RF period^1^. In Flippen (1965)^3^ the time for flux penetration was measured to be between 18 and 28 $\upmu$s for 0.85 mm of penetration. The penetration speed is therefore between 31 and 47 nm/ns assuming instant nucleation. This suggests that the time it takes for flux to enter in a depth comparable to the penetration depth is of the same order of magnitude as the RF period assuming instant nucleation and propagation at constant speed. No data is available which has measured the time for flux to nucleate and penetrate a depth comparable to the RF layer. It is therefore still an open question whether RF cavites can remain in a metastable state above H$_{c1}$ due to the finite time required to nucleate fluxoids. Assuming time scales are not relevant, an increase in $H_{vp}$ due to a present interface energy barrier would be observable in a DC experiment, where $H_{vp}$ can be measured without the effect of RF heating.

Currently, accelerating cavities are made out of bulk Nb due to having the largest critical temperature (T$_{c}$) of any element^1^ and the largest H$_{c1}$ for any known superconductor. For clean Nb, T$_{c}$ = 9.25 K and $\mu _0$H$_{c1}$ is approximately 174 mT at 0 K^4,5^. Using the Ginzburg–Landau parameter, $\kappa _{GL}$, a relation between H$_{sh}$ and the thermodynamic critical field (H$_{c}$) can be made. The Ginzburg–Landau parameter is given by $\kappa _{GL} = \lambda _{L} / \xi$^1^, where for clean Nb $\kappa _{GL} \approx 1$ and $H_{sh} \approx 1.2 H_{c}$. Therefore, for Nb with $\mu _{0}$H$_{c}$ $\approx$ 199.3 ± 10 mT at 0 K^5^, $H_{sh}$ can persist up to $\approx 240$ mT at 0 K. More detailed calculations within Ginsburg–Landau theory can be found in^6^.

To reach large accelerating gradients it is standard practice to perform a low temperature bake (LTB) as a final preparation step. A LTB consists of heating the cavity to 120 $^{\circ }$C for 48 h in ultra-high vacuum^7^. Recently a modified two-step baking process where the cavity is initially baked at a reduced temperature of 75 $^{\circ }$C for 4 h has shown to yield even larger accelerating gradients^8^. Another LTB method which yields accelerating gradients and quality factors in excess to what can be obtained with LTB at 120 $^{\circ }$C is called low temperature nitrogen infusion, often simply referred to as nitrogen infusion. Nitrogen infusion is performed by heating the cavity to 800 $^{\circ }$C in high vacuum for 3 h for H degassing and to separate any Nb$_2$O5, after which the cavity is cooled and held between 120 and 200 $^{\circ }$C with a pressure of 25 mTorr of N^9^. The best Nb cavities prepared by LTB reach a maximum E$_{acc}$ of around 50 MV/m whilst operating at 2 K, which corresponds to a maximum surface magnetic field of $\approx$ 200 mT^10^. This is above $\mu _0$H$_{c1}$ for Nb at 2 K and approximately 10$\%$ lower than the the H$_{sh}$ which is the expected maximum value.

The increased surface magnetic field due to LTB techniques can be due to a number of possibilities. The baking could eliminate the cause of the high field Q-slope for example by avoiding the growth of dissipative niobium hydrides^11^. The change in penetration depth^12^ results in a reduced surface current. This could have the effect that localized quenching and dissipation is delayed or completely prevented^13^. Finally, the dirty layer could introduce an interface energy barrier for flux penetration from the dirty layer to the bulk of the material^14^. Depending on the layer thickness it is possible that there might only be two distinct energy barriers for a sufficient dirty layer thickness. Calculations from Checchin suggest that the layer thickness should be on the order of 60 nm^15^ comparable to what LE-muSR studies suggest. In this paper the focus is on the interface barrier. The aim of this study is to determine if an interface barrier is present within a LTB sample, and hence an increased H$_{vp}$ due to a change in the surface layers produced by the LTB procedure.

It is known that all LTB processes described above yield a larger penetration depth and therefore a reduced screening current in the outer layer exposed to the RF field. Low energy muon spin rotation results have shown that there is a strong change in Meissner screening at a depth of about 60 nm for 120 $^{\circ }$C baked niobium^12^. This could yield a superconductor–superconductor (SS) interface energy barrier for flux penetration at the boundary between the dirty layer and the clean bulk superconductor similar to the Bean-Livingston barrier at the superconductor-vacuum interface^14^ delaying flux penetration and therefore increasing the field of first vortex penetration ($H_{vp}$). Junginger et al.^16^ have argued that in the presence of defects only the interface barrier can prevent flux penetration as the order parameter can be restored in the vicinity of the defects at the interface but not at the boundary. It should be noted that their study focused on actual bilayers composed of two different superconductors. Their results on low temperature baked niobium showed only a small H$_{vp}$ increase which might be due to surface pinning.

### Sample testing

The aim of this study is to test with DC magnetometry whether the increase in accelerating gradient caused by different LTB processes can be correlated to an increased DC field of first vortex penetration. High temperature annealed ellipsoidal samples were used to avoid edge and pinning effects. For details on the preparation see the method section. Four samples were tested. One received no further heat treatment after annealing, whilst the other three were subject to a LTB. The samples were tested in a SQUID magnetometer, specifically a Quantum Design MPMS 3. The field applied by a SQUID magnetometer is from a solenoid much larger than the sample itself, and the sample is positioned inside the solenoid such that the applied field is uniform. With an ellipsoidal sample, the flux lines around the ellipsoid will be denser around the equator of the ellipsoid, and therefore the local field on the sample surface is larger than H$_{ext}$. The demagnetization factor *N* relates the field at the equator $H_{eq}$ to $H_{ext}$ by $H_{eq} = H_{ext} / (1 - N)$^17^, where N = 0.13 for the ellipsoidal samples used in this study. The magnetometer is ideally suited for samples of length shorter than 5 mm due to the size of the pick-up solenoids^18^.For longer samples the magnetic moment will be underestimated. Our samples are 10 mm long. The expected magnetic moment for a perfect diamagnet assuming a demagnetization factor of N = 0.13 would be about 20$\%$ higher than the data obtained for the irreversible magnetization curve.

Generally samples were zero field cooled (ZFC) for each 5 quadrant hysteresis loop measurement at fixed temperature. The reported H$_{ext}$ is determined by the current known to be passing through the solenoid which applies the field, and the applied field could be different due to the history of the magnet as flux could be trapped within the solenoid^18^. Therefore after each hysteresis run the magnet was de-gaussed to reduce pinning in the magnet, before the sample was heated above T$_{c}$ to remove any pinning from the sample. The samples were then warmed up, and held at 12 K for 5 min to expel any flux that could be trapped within the sample, before undergoing ZFC again. As the external field H$_{ext}$ is swept it does not stabilise to a specific value so the reported H$_{ext}$ are averages^18^.

**Figure 1:**
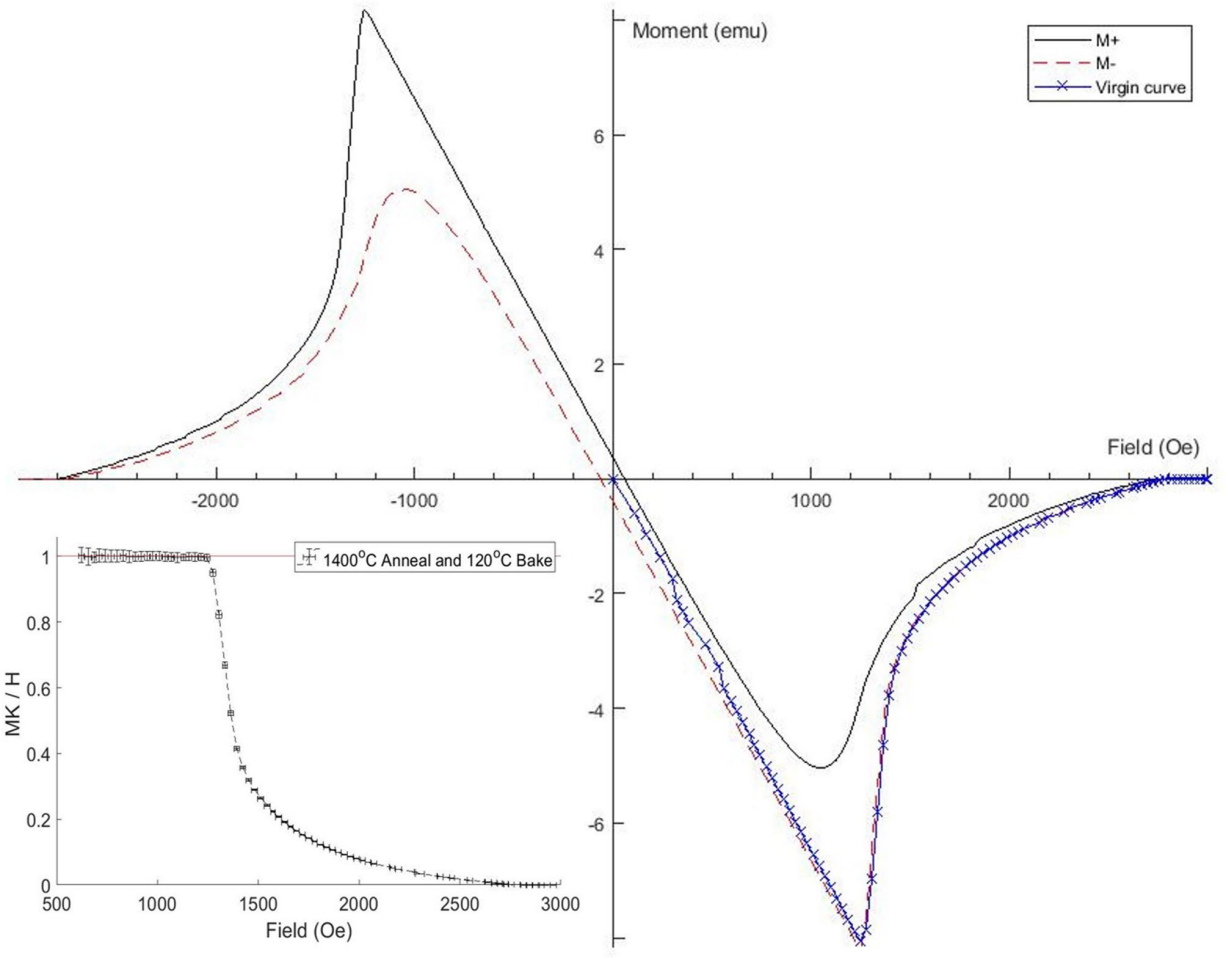
Hysteresis loop for the 120 $^{\circ }$C baked ellipsoid. The initial increase and decrease in the externally applied field is known as the virgin curve, shown in blue. The positive and negative moment used to determine pinning strength are shown in red and black. The standardisation curve used to determine H$_{vp}$ is shown in the bottom left quadrant, which is determined using the virgin curve. The last point within error of 1 is taken as H$_{vp}$.

Each testing cycle begins at $\mu _0$H$_{ext}$ = 0 mT, such that there is no magnetic moment produced. The external field is then slowly increased which results in a perfect diamagnetic response produced from the superconductor, which is shown in Fig. 1 by the initial curve (straight line in fourth quadrant starting from the origin). It can be observed that as H$_{ext}$ is initially increased, the resulting moment is not perfectly linear. This has been observed for each sample. When the local field on the surface of the superconductor reaches H$_{vp}$ the flux enters the superconductor dividing the ellipse into normal conducting/superconducting regions. Once the vortices have entered the sample, the superconductor has transitioned from the Meissner state to the Abrikosov state and the response of the magnetic moment to the applied field is no longer linear. This is due to more vortices penetrating into the superconductor, in turn reducing the superconducting volume. As H$_{ext}$ continues to be increased, the moment increases until H$_{c2}$, where the moment becomes slightly positive due to the paramagnetic response of the normal conducting Nb. The external field is then decreased. By decreasing the field, the flux is then expelled from the superconductor, and the magnetic moment becomes negative again. In the case of a perfect superconductor, the produced magnetic moment would be the same for both increasing and decreasing H$_{ext}$. It can be seen in Fig. 1 that this is not the case here. The absolute value of the magnetic moment is smaller than for the initial curve. This is due to trapped flux within the sample.

After H$_{ext}$ has reached zero again the field is ramped at a faster rate with reversed polarity (negative applied field). These results are shown by the black and red curves in Fig. 1.

Each hysteresis cycle ends with a repetition of the initial virgin curve. This is also done to ensure that the sample has not moved during the test.

### Determining the field of first flux penetration

To determine the field of first flux penetration, only the initial curve produced by increasing H$_{ext}$ is used as there is no magnetic history which can affect the results. Whilst in the Meissner state the response of the superconductor is linear due to H$_{ext}$, and can be described as $M = K^* H_{ext}$^19^, where M is the magnetic moment and $K^*$ is a constant proportional to the superconducting volume, which can vary slightly between samples. By normalising $MK^{*}/H$ in the Meissner state to 1 as shown in the bottom left quadrant of Fig. 1, H$_{vp}$ can be determined by the last point to be within error of 1. Once H$_{ext}$ has been found, the geometry of the sample must be taken into account. Due to the geometry of the ellipsoid, N is 0.13, such that $H_{vp} = 0.87 H_{ext}$. This method is done for each sample at each temperature.

### Determining irreversible pinning strength

**Figure 2:**
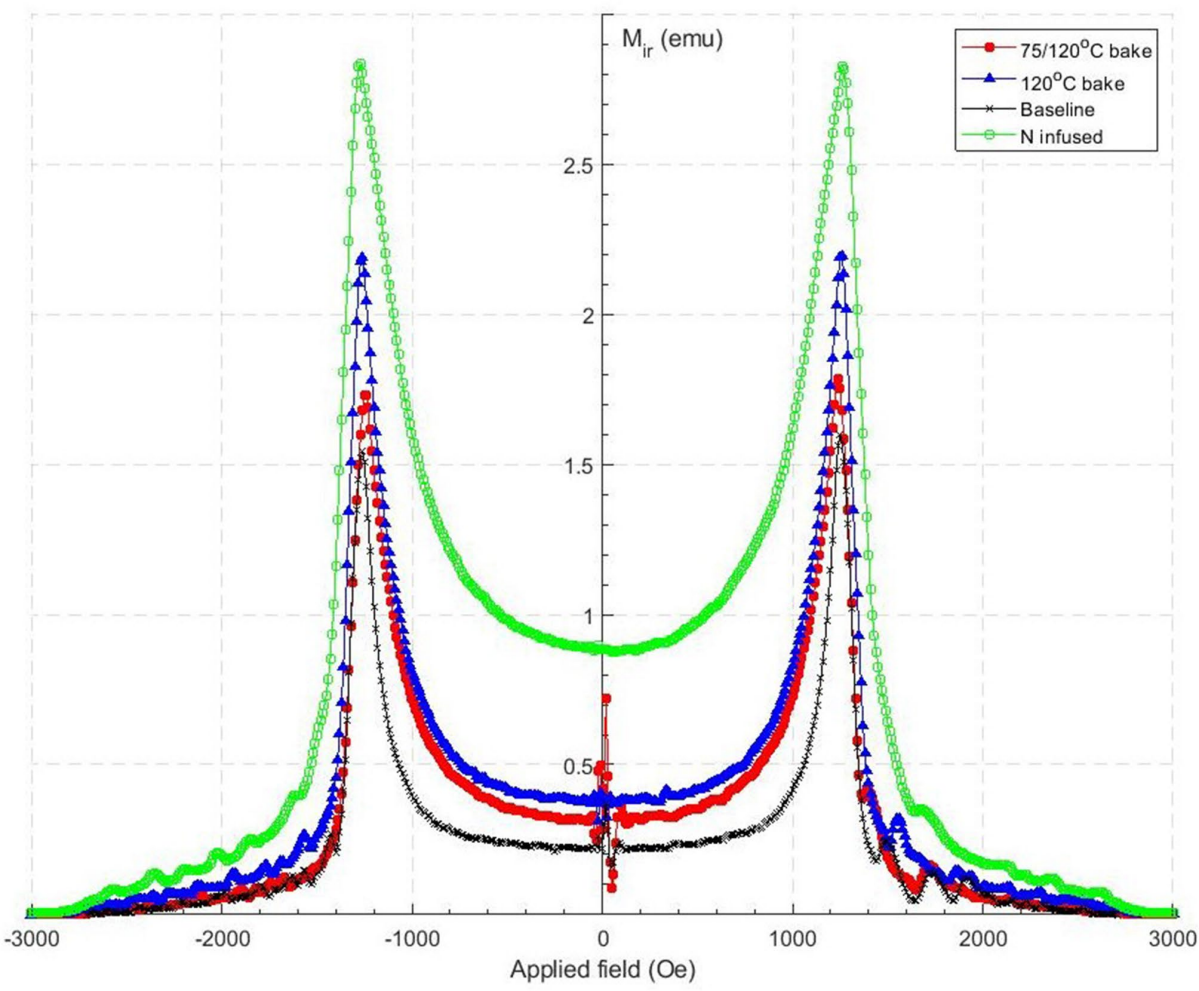
The irreversible pinning (M$_{ir}$) for each treatment at 4.2 K.

In an ideal pin-free superconductor once H$_{ext}$ has increased above H$_{c2}$ and is then decreased, the magnetic moment produced by the sample is identical to the initial magnetisation loop. If the superconductor is not pin-free, the return loop for the magnetisation curve will differ, which is found in all the hysteresis graphs presented in this paper. To determine the pinning strength produced by each treatment the irreversible magnetization was calculated using the hysteresis loops shown in Fig. 1, using both the positive and negative moment. The irreversible magnetization is then found using $M_{ir} = (M^+ - M^-)/2$^20^, with both M$^+$ and M$^-$ shown in Fig. 1. The M$_{ir}$ is plotted as a function of H$_{ext}$ for each sample at 4.2 K in Fig. 2. The M$_{ir}$ is the largest at H$_{vp}$ where the return loop does not follow the initial curve due to pinning within the sample. The pinning strength (M$_{pin}$) for each temperature and treatment is then taken at the point where $\mu _{0}$H$_{ext}$ is 0 mT, ie M$_{ir}$(0 Oe) = M$_{pin}$, as the magnetic moment is being produced by the sample is not a response to a H$_{ext}$. The irreversible pinning for each treatment is shown in Table 1.

## Results

**Figure 3:**
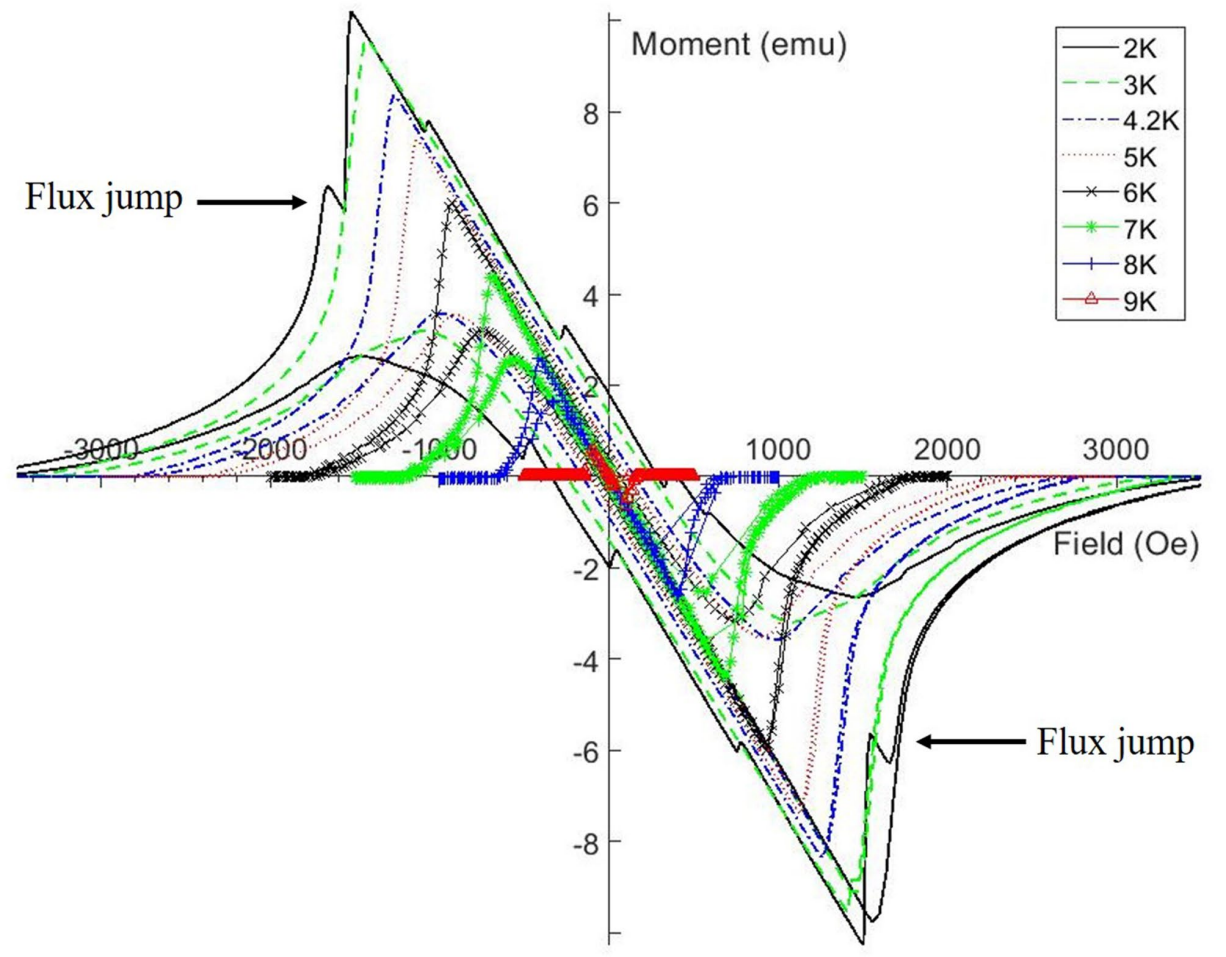
The hysteresis loops performed on the N infused sample at varying temperatures. Flux jumps can be seen once the sample had been taken above H$_{c2}$ for the 2 K data only.

A hysteresis loop was performed at 2, 3, 4.2, 5, 6, 7, 8, and 9 K for all samples except the 120 $^{\circ }$C baked one. The effect of temperature on the hysteresis loops can be seen in Fig. 3, where the increasing temperature reduces the critical fields of the superconductors. It should be noted that the hysteresis loops for the Baseline, 120 $^{\circ }$C bake and the 75/120 $^{\circ }$C bake have similar looking hysteresis curves across all temperatures respectively. I.e. each sample experiences smooth transitions as H$_{ext}$ varies. This is not the case for the N infused sample. It can be seen that after the sample had been increased above H$_{c2}$, the moment has some sharp transitions shown in the top left quadrant and the bottom right quadrant (indicated by the arrows) and in low H$_{ext}$ shown in Fig. 3. These flux jumps are only visible at 2 K. These sharp transitions indicate flux jumps where trapped flux suddenly moves within the sample, from one pinning center to another due to a change of forces as H$_{ext}$ is increased and more vortices enter the ellipse. This only happens after the ellipse had already been taken to H$_{c2}$ to take the superconductor into the normal conducting regime. There is no flux jump at 2 K when H$_{ext}$ is initially increased and decreased which allows us to determine that the flux has been trapped after the sample was in the normal conducting state with H$_{ext}$ > H$_{c2}$.

**Figure 4:**
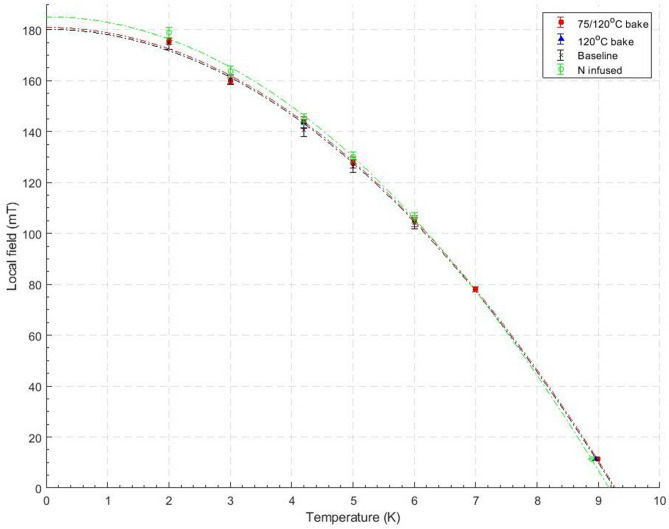
The field of first flux penetration as a function of temperature for all 4 samples. The line of best fit is shown for each sample except the 120 $^{\circ }$C.

The field of first vortex penetration was found for each sample at various temperatures by using the standardisation curve method described above. Once H$_{vp}$ was found and the field enhancement accounted for by the demagnetization factor, a graph of H$_{vp}$ as a function of temperature could be plotted, shown in Fig. 4.

It was found that H$_{vp}$ fits the expression $H_{vp}(T)=H_{vp}(0)(1-(T/T_{c})^2)$ allowing extrapolation to determine H$_{vp}$ at 0 K, as well as extrapolating to T$_c$ when H$_{vp}$ = 0 mT. It can be seen from Fig. 4 and Table 2 that there is no significant change in H$_{vp}$ produced by low temperature baking or N-infusion when tested in DC magnetometry. In addition it can be seen in Table 1 that there is no change between extrapolated critical temperature between samples.

**Table 1:** The field of full flux penetration for each set temperature and the critical temperature determined by using the linear dependence of H$_{vp}$ vs T$^{2}$ (T$_c$ (0 mT)), assuming a linear T$^{2}$ dependence.

T, K	$\mu _{0}$H$_{vp}$(T), mT for each treatment			
	Baseline	120 $^{\circ }$C bake	75/120 $^{\circ }$C bake	N infusion
2	174.6 ± 2.19	–	175.5 ± 1.21	179.0 ± 2.06
3	160.5 ± 1.84	–	159.9 ± 1.09	163.7 ± 2.07
4.2	140.7 ± 2.64	143.1 ± 1.55	143.4 ± 2.30	144.7 ± 2.18
5	126.3 ± 2.41	–	127.7 ± 2.18	129.9 ± 2.07
6	104.0 ± 2.18	–	104.7 ± 2.19	106.16 ± 2.07
7	–	–	78.0 ± 1.09	–
T$_c$ (0 mT)	9.24 ± 0.01	–	9.24 ± 0.01	9.17 ± 0.01

**Figure 5:**
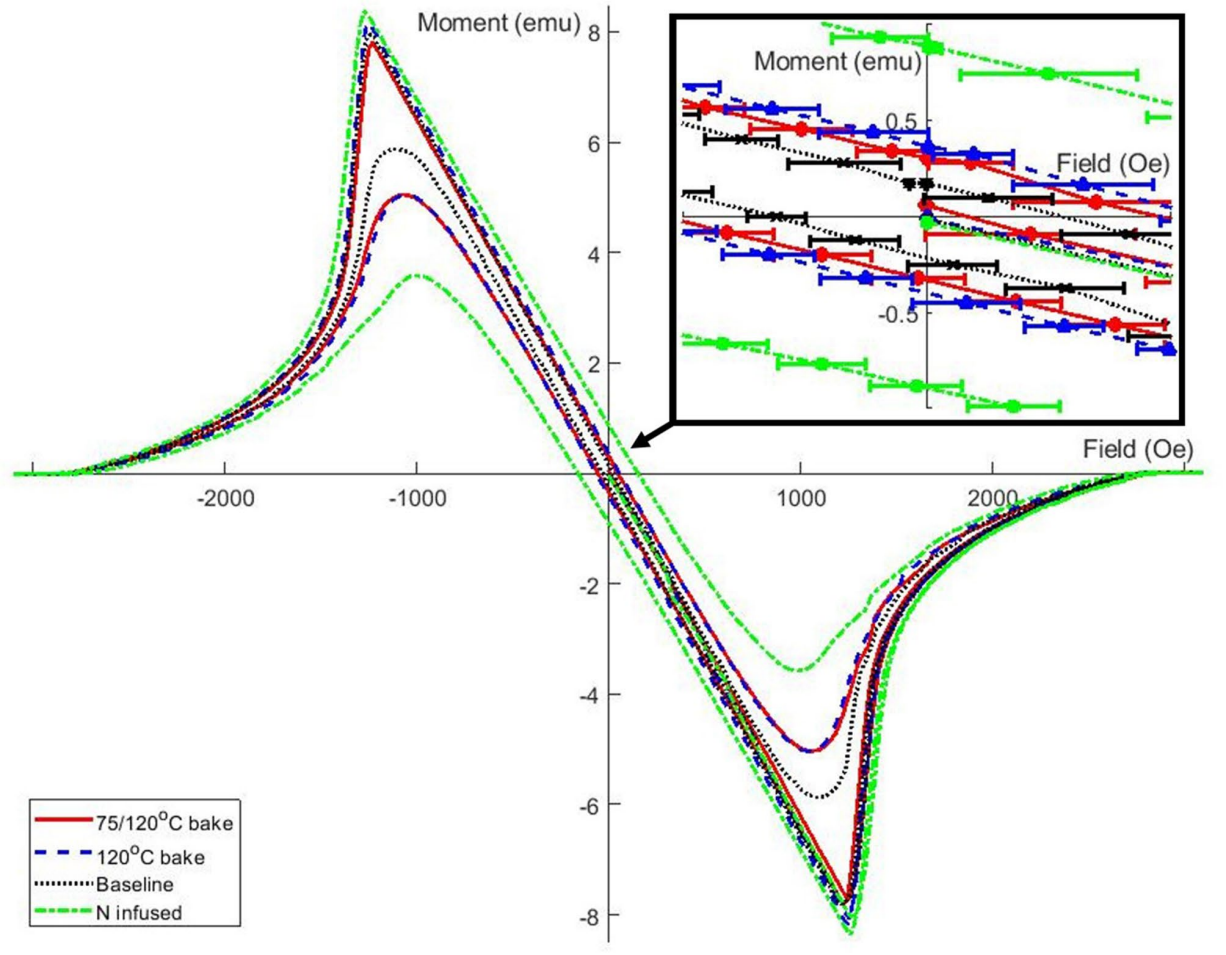
The hysteresis loops at 4.2 K for all four samples, with a magnified image in the top right for the residual moment when the μ_0_H$_{ext}$ = 0 mT.

An interesting difference between the four samples is their pinning strength as can be seen in the inset in Fig. 5 and Table 2.

The difference in magnetic moment for increasing to decreasing H$_{ext}$ is indicative of the pinning strength. A pin free sample would yield zero magnetic moment for H$_{ext}$=0 in both cases. The baseline sample has the weakest pinning. The pinning strength for the 120 $^{\circ }$C and 75/120 $^{\circ }$C samples is larger and very similar, while nitrogen infusion yields even stronger pinning.

**Table 2:** Irreversible magnetic moment obtained at $\mu _{0}H_{ext}$ = 0 mT indicative of the pinning strength.

T, K	M$_{ir}$, emu for each treatment			
	Baseline	120 $^{\circ }$C bake	75/120 $^{\circ }$C	N infusion
2	0.44 ± 0.016	–	0.82 ± 0.0076	1.9 ± 0.0063
3	0.24 ± 0.0063	–	0.50 ± 0.0071	1.7 ± 0.0085
4	0.22 ± 0.0058	0.39 ± 0.0090	0.2847 ± 0.013	0.88 ± 0.0051
5	0.94 ± 0.0075	–	0.18 ± 0.0057	0.64 ± 0.0056
6	0.082 ± 0.0084	–	0.14 ± 0.0055	0.37 ± 0.0058
7	0.076 ± 0.0058	–	0.067 ± 0.0066	0.19 ± 0.0058
8	–	–	0.035 ± 0.0055	0.055 ± 0.0055

## Discussion

Four Nb ellipsoids were machined and then annealed to eliminate pinning within the samples to produced accurate results when tested using DC magnetometry. Three of the four samples saw further LTB treatments. The samples were held at a set temperature before a hysteresis loop was measured, from which H$_{vp}$ was determined taking the well defined demagnetization factor into account. The H$_{vp}$(T) for each sample is shown in Table 1 and Fig. 4. Interpolating results to 0 K yields $\mu _{0}H_{vp}$(0 K) = 179.9 mT for the baseline sample. This is comparable to previous measurements using muon spin rotation^4^ of $\mu _0$H$_{c1}$ = 174 mT and magnetometry 173.5 mT^5^. No significant $H_{vp}$ increase was observed for all LTB samples.

This shows that the LTB processes do not yield an interface barrier for flux penetration, at least in the DC case. This is different to results obtained for bilayers of MgB$_2$ and Nb$_3$Sn on niobium. Tan et al.^21^ found that 200 nm of MgB$_2$ on Nb increased the field of first flux penetration by approximately 40 mT compared to uncoated niobium^21^ using a MPMS SQUID magnetometer in a similar experiment to the one presented here, where it has been shown that a second superconducting material on the surface can delay H$_{vp}$ into the bulk of the sample. It could be possible that using these methods, an increase in H$_{vp}$ could be attributed to the flux being pinned in the surface layers, which cannot be differentiated from an interface barrier being present. It is possible that the pinned flux can be due to alternative mechanisms other than the surface barrier, such as a surface sheath^22^ and surface flux pinning^23^. As there is no increase in H$_{vp}$, we can verify there is no mechanisms which interfere with H$_{vp}$, either the interface barrier or surface pinning. These results are consistent with muon spin rotation ($\upmu$SR) experiments performed on MgB$_2$ and Nb$_3$Sn on niobium samples^16^. These studies suggest an increase in H$_{vp}$ from a field consistent with H$_{c1}$ to a field consistent with H$_{sh}$ of clean niobium due to the overlayer. These results showed no significant dependence on layer thickness (50–3000 nm were tested), therefore suggesting that it is indeed the interface barrier which causes the increase in H$_{vp}$. This study also found a slight increase of $\mu _{0}$H$_{vp}$ from 178 mT to 188 mT for 120 $^{\circ }$C baked niobium. This effect can potentially be related to surface pinning in a layer thinner the implantation depth of the muons of about 0.15 mm. It should be noted that the effect of the interface barrier in LTB cavities might still be relevant for time-varying RF field. However, the comparison of DC studies on LTB niobium and actual bi-layer samples suggest that this effect is only relevant for actual bi-layers composed of two distinct superconductors.

Alternative methods have been used to determine H$_{vp}$ to determine if there is any difference between methods. The alternate method has been presented by Roy et al.^24^, using the square root of the deviation of the magnetic moment from a straight line as a function of H$_{ext}$. This technique agrees with the aforementioned technique presented by Wilde et al.^19^ with a deviation in H$_{vp}$ up to 5$\%$, depending on temperature and baking technique. The two techniques to determine H$_{vp}$ produced no significant change.

Roy et al. found 2 slopes using the square root of the deviation method^24^, and determined the slopes to be either the Bean–Livingston surface barrier or an effect of geometry. The square root of the deviation for the LTB ellipsoids presented in this paper do not show two slopes as presented by Roy et al., such that it can be concluded that the two slopes must be geometrical factors. These slopes are presented in the Supplementary Information for 4.2 K for each ellipse.

A measurable effect produced by each treatment is the amount of flux pinning in each sample, shown in Fig. 5. The baseline sample had the least amount of trapped flux, as shown by its magnetic moment once $\mu _{0}$H$_{ext}$ had returned to 0 mT. The low temperature bake samples then had the next greatest moment, and finally the N infused samples had an even larger moment when $\mu _{0}$H$_{ext}$ had been reduced back to 0 mT. Based on this result one can argue that pinning of flux in the outer surface layer is a possible explanation for the delayed high field Q-Slope onset at low temperature.

The pinning results agree with measurements performed by Furtado^25^, in which Nb cylinders were annealed, followed by mechanical and chemical polishing, and finally pulled. The treatments performed affected the surface of the Nb cylinders. The results stated that the condition of the surface is the main factor for increased surface pinning, as mechanical polishing increased the amount of flux trapped within the sample, however a further buffer chemical polish (after machining) removed the flux pinning (Fig. 2).

For reference niobium cavities treated by EP have a HFQS onset at $\approx$ 100 mT^13^. Low temperature N infusion of a cavity has found to delay the onset of the HFQS until the peak magnetic field on the cavity walls is $\approx$ 190 mT^13^. It has also been found that subsequent removal of the surface of the cavity by HF rinse returns the high field Q slope to its previous level, therefore concluding that N infusion only affects a few nanometers on the surface of the sample^13^. The change in the amount of pinning between all four ellipsoid must be attributed to changes on the surface of the material.

If the surface of the thickness of the dirty layer produced by LTB is too thin, a nascent vortex can act as a nucleation site for the magnetic flux to enter the superconducting sample^26^. It has been suggested that an effective depth for a bilayer is 60 nm^15^. Probing the surface layers using LE-$\upmu$SR determines that LTB changes the magnetic profile up to 60 nm^12^, however an increase in H$_{vp}$ has been determined experimentally for bilayers consisting of 50 nm of MgB$_2$^16^.

In conclusion the results presented here suggest that the delayed HFQS onset might be due to efficient pinning of penetrating vortices in the outer surface layer. Our measurements and comparison with data on actual bilayer samples suggest that LTB does not yield and effective interface energy barrier, however experiments^16,21^ suggest a dirty layer in the same order of thickness should be possible to create a barrier. There are other potential mechanisms which are neither supported by or in contradiction to our results, which may lead to an explanation for SRF cavities reaching magnetic fields above H$_{c1}$. These include reduced RF heating due to a reduction of the surface current and mechanisms which suggest the removal of the cause for the HFQS such as^11^, or effective pinning of vortices in the dirty layer. Further studies should focus on understanding the influence of reduced surface current and pinning on the HFQS.

## Methods

Each ellipsoid was hand polished to remove the edges produced by machining, followed by buffer chemical polishing (BCP) to remove any damaged layers. Following this, the ellipsoids were annealed for 5 h at 1400 $^{\circ }$C to remove stresses within the Nb that were present before the machining or produced during machining. This process has shown to remove virtually all pinning^4^. Finally, the ellipsoids had a final round of BCP (10 $\upmu$m) to remove any contaminants that could have been introduced from the oven. One ellipsoid saw no further treatment, and was used as a baseline sample to compare all further treatments too, and is referred to throughout the paper as baseline,which weighs 768.4 mg. One ellipsoid was baked at 120 $^{\circ }$C for 48h, and another sample was baked at 75 $^{\circ }$C for 5 h followed by a 120 $^{\circ }$C bake for 48 h which are referenced throughout the paper as 120 $^{\circ }$C and 75/120 $^{\circ }$C, respectively, which weigh 768.2 and 772.7 mg respectfully. Finally, a sample was sent to FNAL for N infusion which includes heating the sample to 800 $^{\circ }$C under vacuum and waiting for 3 h under high vacuum, followed by reducing the temperature to 120 $^{\circ }$C and 25 mT of N is injected into the furnace and maintained at this pressure and temperature for 48 h^9^. This sample is labelled as the N infused ellipse, and weighs 770.4 mg. Each sample was made to be 10 mm tip to tip of the ellipsoid with a 4 mm diameter at the equator.

## Supplementary Information

Supplementary Information.
