# Supplementary material for: No interface energy barrier and increased surface pinning in low temperature baked niobium
Source: Sci Rep. 2022 Apr 1;12:5522. doi: 10.1038/s41598-022-09023-0 (PMC8976006; doi:10.1038/s41598-022-09023-0)
Supplement: Supplementary file 1 — Supplementary Information. [file 41598_2022_9023_MOESM1_ESM.pdf]

Supplementary Material

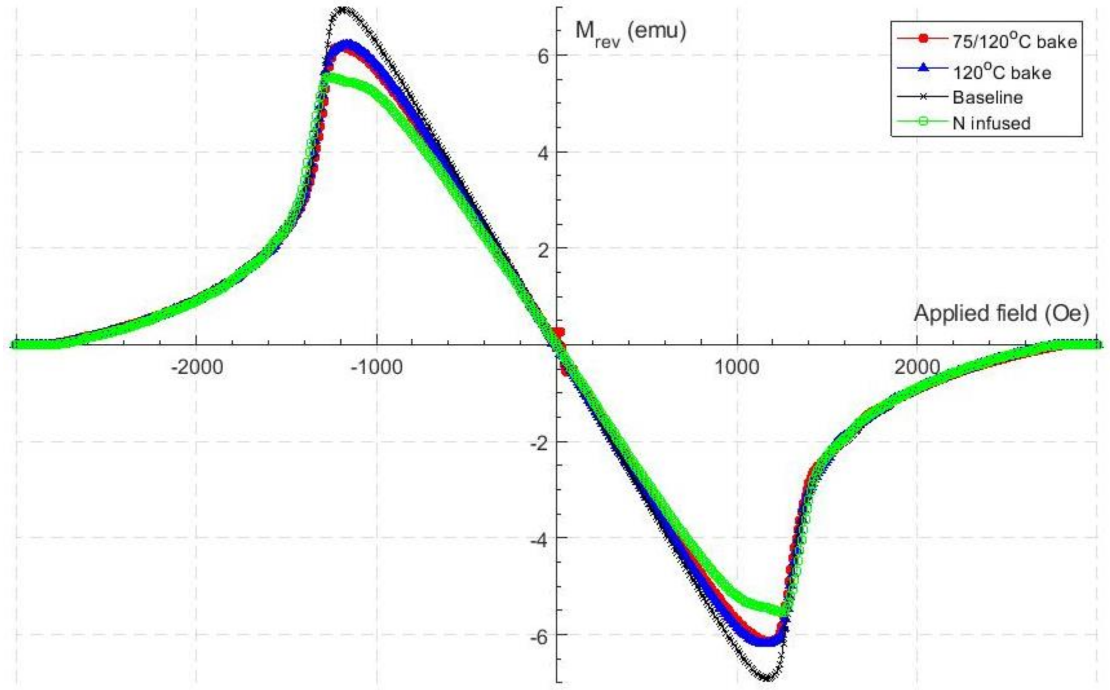

Figure 1 – The reversible magnetisation curve for each sample at 4.2 K, taken using the equation $M_{rev}=(M^{+}+M^{-})/2$.

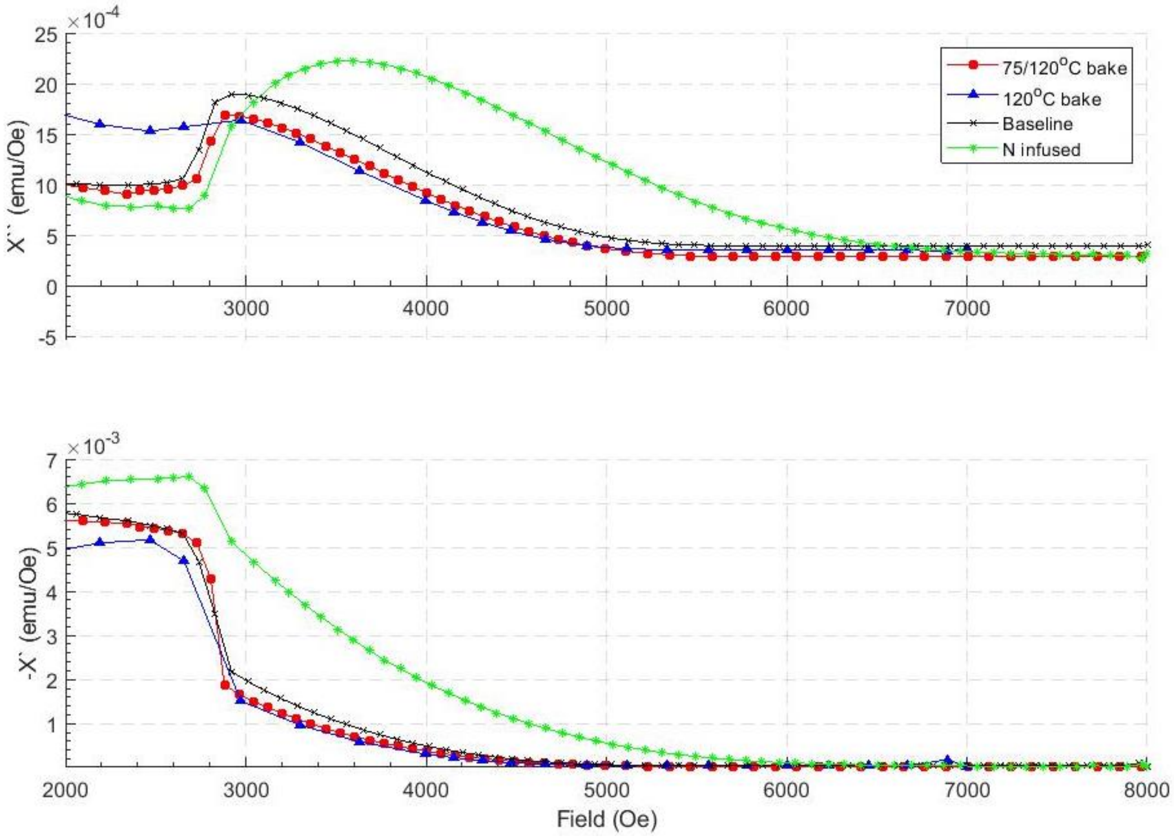

Figure 2 – The imaginary and real component of the AC susceptibility for each ellipsoid produced, performed at 4.2 K and 10 Hz. There is a clear increase in H_{c3} for the N infused sample compared to other LTB samples. The LTB samples also show no clear increase from the baseline sample.

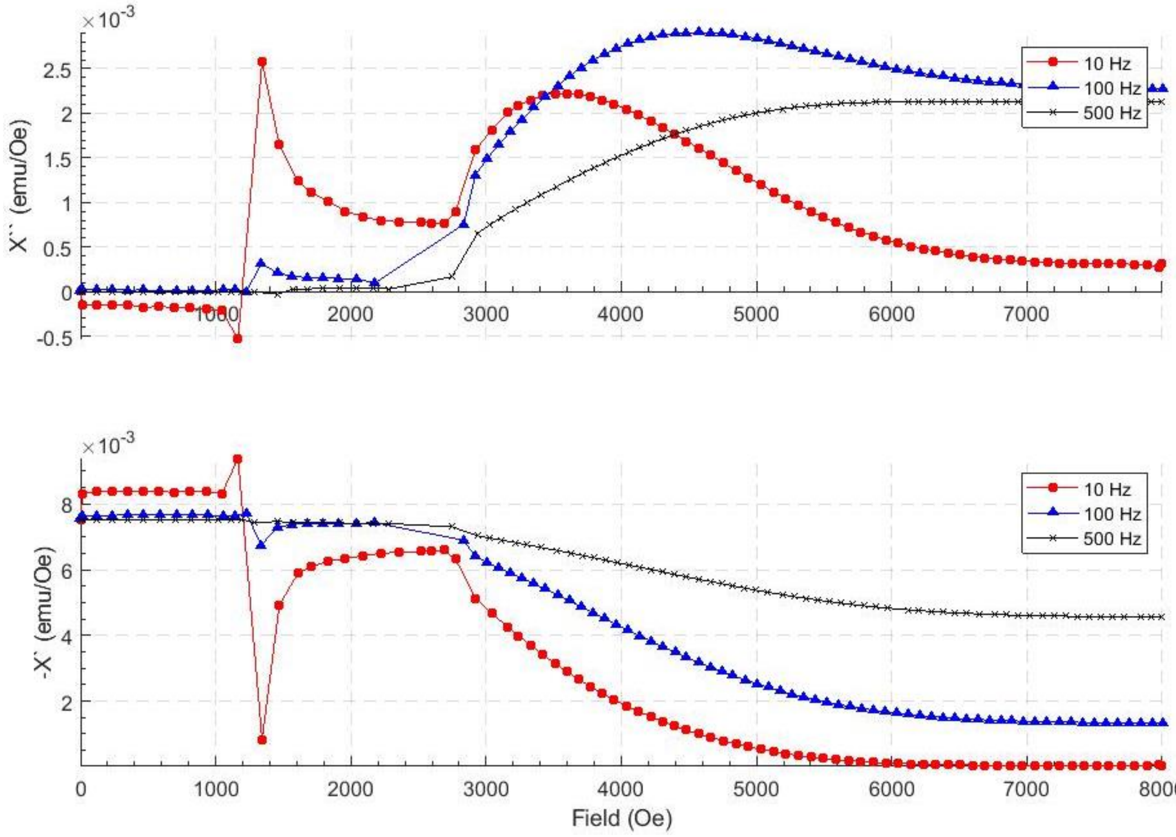

Figure 3 – AC susceptibility measurements were performed at 3 different frequencies on the N infused sample to try and determine which frequency would be best to investigate the surface layers of the ellipse.

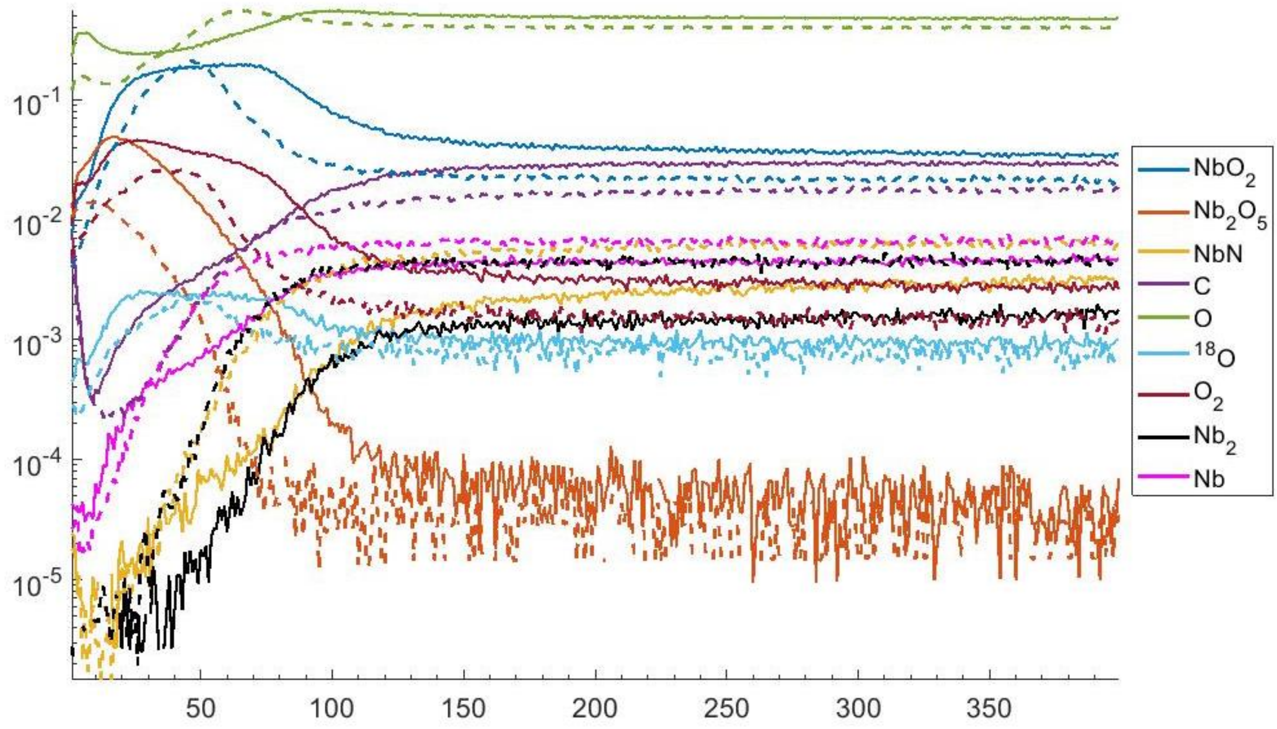

Figure 4 –SIMS data of the baseline ellipse (dashed line) and the N infused ellipse (solid line). The NbO2 signal can be used as a reference for the length scale.

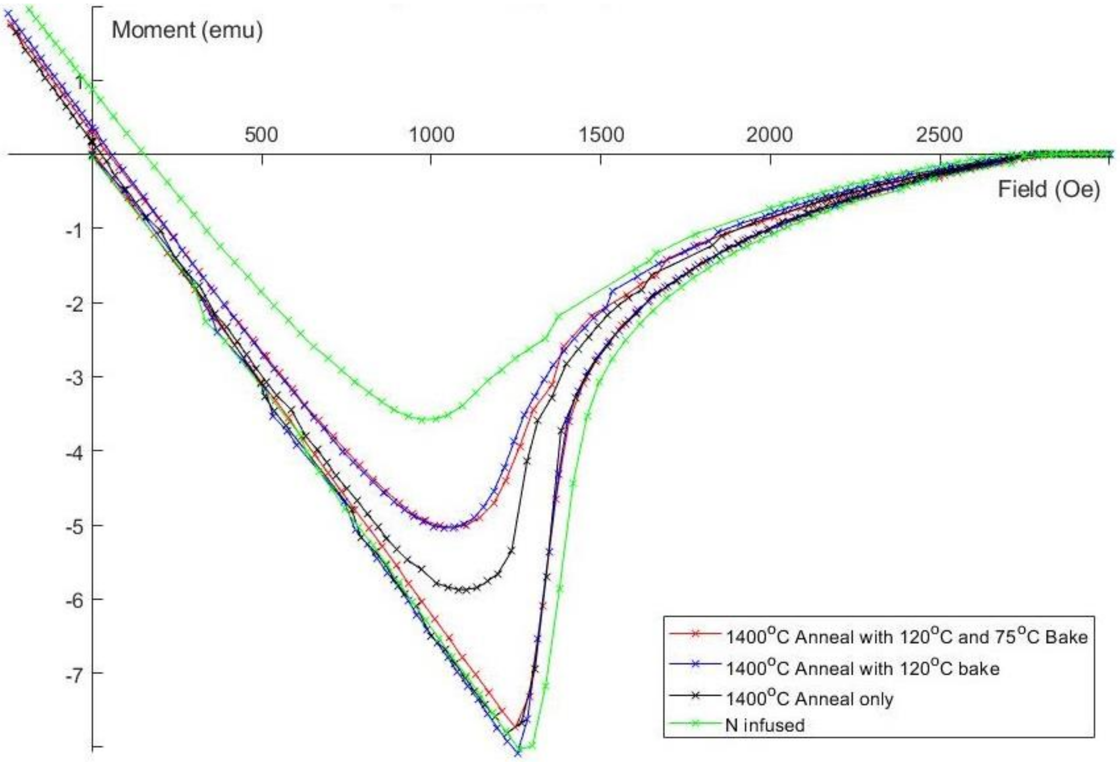

Figure 5 – The first quadrant of the hysteresis for each ellipsoid. The initial increase in the magnetic moment is not perfectly linear, occurring for each ellipse. This could be due to the type of measurements taken and would explain the systematic pattern in results at low H_{ext}.

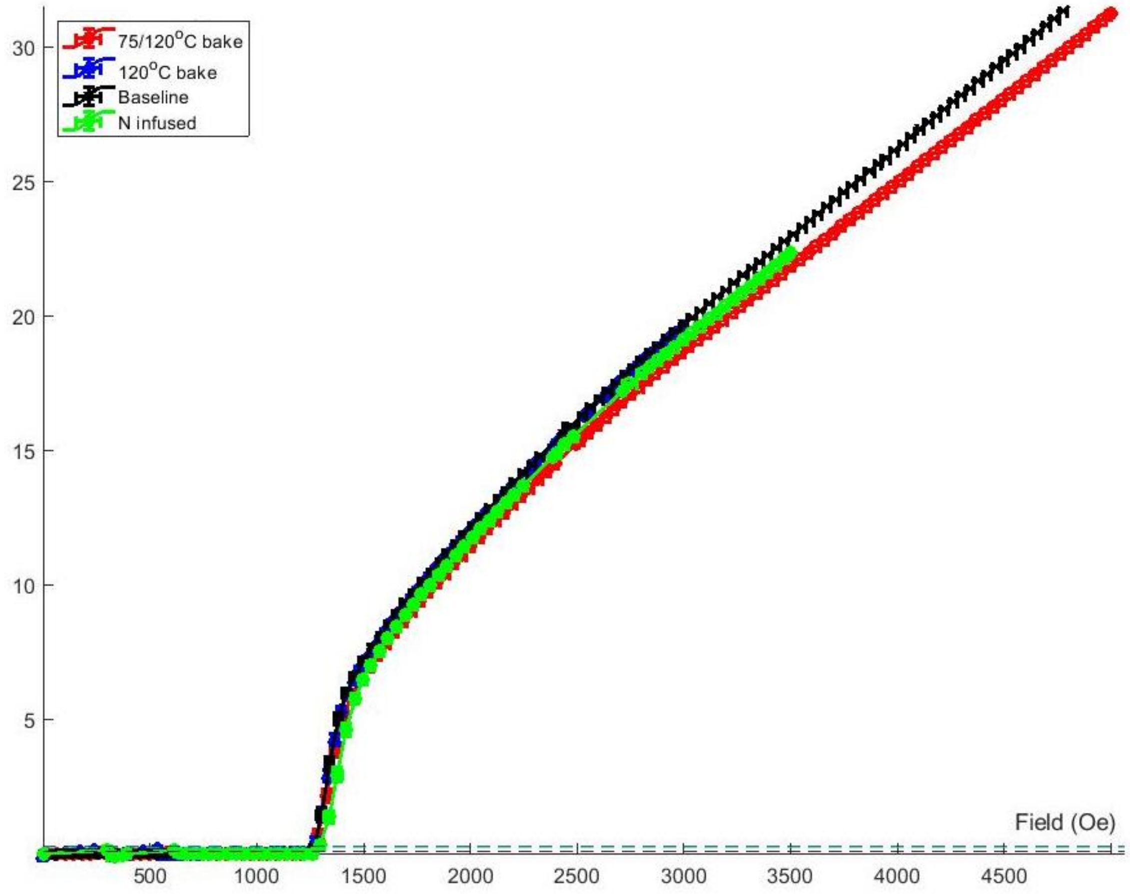

Figure 6 – Square root of the deviation of magnetization from a linear field dependence in the Meissner state for each sample. The dashed lines parallel to the x-axis (Field) is the threshold for H_{vp}. This is the standard deviation method presented in Roy, Myneni, and Sahni, Supercond. Sci. Technol 21, 065002 (2008).

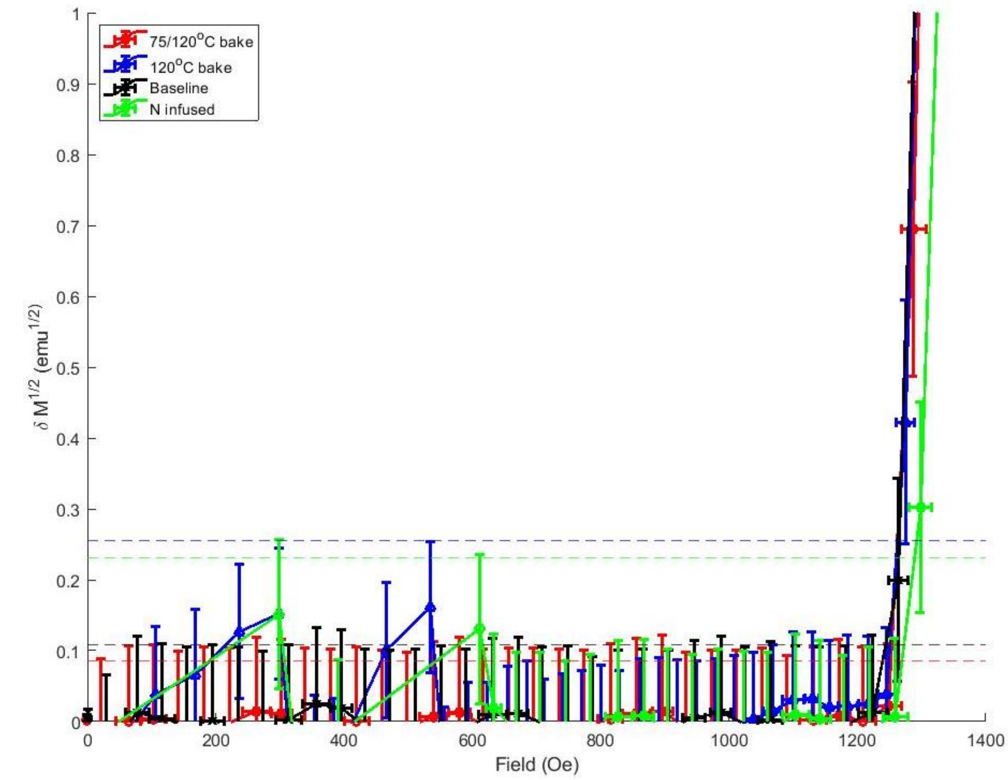

Figure 7 – An increased magnification on the point of H_{vp} using the square root of the deviation of magnetization presented in Roy, Myneni, and Sahni, Supercond. Sci. Technol 21, 065002 (2008).

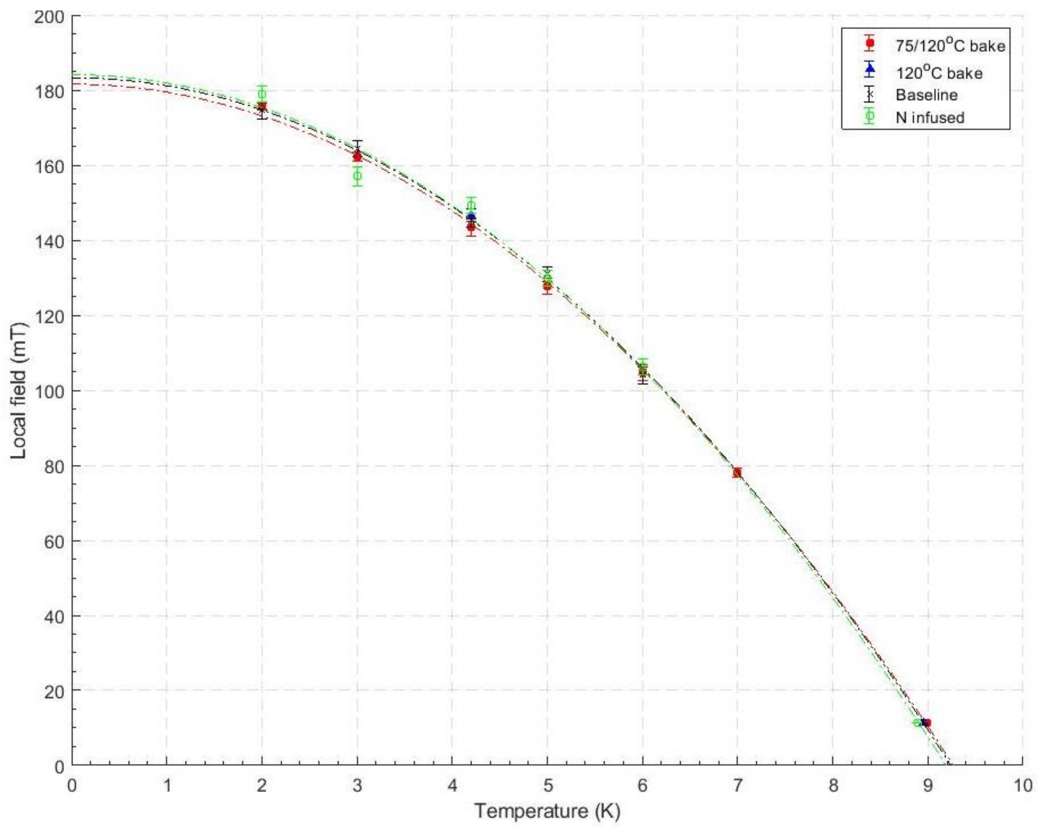

Figure 8 – The field of first flux entry as a function of temperature, where the field of first flux entry has been found using the method presented in Roy, Myneni, and Sahni, Supercond. Sci. Technol 21, 065002 (2008). The values plotted are also shown in the table below.

	$\mu_0 H_{vp}(T)$, mT for each treatment			
**T [K]**	**Baseline**	**120°C bake**	**75/120°C bake**	**N infusion**
**2**	1794.6 ± 2.2		175.5 ± 0.9	179.0 ± 2.1
**3**	164.5 ± 1.9		162.2 ± 1.2	157.0 ± 2.5
**4.2**	149.2 ± 1.7	146.6 ± 1.6	143.4 ± 2.0	149.2 ± 2.1
**5**	130.9 ± 2.0		127.7 ± 2.2	129.9 ± 2.1
**6**	104.0 ± 2.2		104.7 ± 2.2	106.2 ± 2.1
**7**	-		78.1 ± 1.1	-
**T_c(0 mT)**	9.24 ± 0.01		9.27 ± 0.01	9.19 ± 0.01

Table 1 – B_{vp} for each temperature and the extrapolated T_c value using a linear fit for T^2. Here B_{fp} was found using the same method as described in Roy, Myneni, and Sahni, Supercond. Sci. Technol 21, 065002 (2008).
